# Acquisition, image quality, and PI-RADS agreement of ultrahigh-gradient DWI in prostate 3-T MRI

**DOI:** 10.1186/s41747-026-00684-4

**Published:** 2026-02-23

**Authors:** Leon M. Bischoff, Christoph Endler, Philipp Krausewitz, Joerg Ellinger, Niklas Klümper, Alexander Isaak, Narine Mesropyan, Dmitrij Kravchenko, Daniel Kuetting, Alois M. Sprinkart, Petra Mürtz, Claus C. Pieper, Julian A. Luetkens

**Affiliations:** 1 Department of Diagnostic and Interventional Radiology, University Hospital Bonn, Bonn, Germany; 2 Quantitative Imaging Lab Bonn (QILaB), University Hospital Bonn, Bonn, Germany; 3 Department of Urology and Pediatric Urology, University Hospital Bonn, Bonn, Germany

**Keywords:** Diffusion magnetic resonance imaging, Multiparametric magnetic resonance imaging, PI-RADS, Prostate, Prostatic neoplasms

## Abstract

**Objective:**

New magnetic resonance imaging (MRI) gradient technology enables the acquisition of ultrahigh *b-*value diffusion-weighted imaging (DWI). We assessed its impact on image quality and Prostate Imaging Reporting and Data System (PI-RADS) scores in prostate MRI.

**Materials and methods:**

Participants with cancer suspicion prospectively underwent 3-T prostate MRI (maximum gradient strength 200 mT/m). Sequences with *b-*values of 0/800, 1,500, 2,500, 3,500, and 4,500 s/mm² were acquired. Lesion conspicuity was rated from 1 (non-diagnostic) to 5 (excellent). Apparent signal-to-noise ratios (aSNR) and acquisition times were determined. Cumulative link mixed-effects models, repeated measures ANOVA, and Cohen/Fleiss κ statistics were used.

**Results:**

A total of 107 participants, aged 67 ± 8 years (mean ± standard deviation), were included. Compared to DWI(b1500), the DWI(b2500), DWI(b3500), and DWI(b4500) acquisitions were worse regarding both lesion conspicuity (median score, 5 [interquartile interval 4–5] *versus* 4 [3–4] *versus* 2 [2–3] *versus* 2 [1–2], respectively; all *p* < 0.001) and aSNR (19.0 ± 7.5 *versus* 12.7 ± 4.8 *versus* 11.8 ± 4.1 *versus* 11.4 ± 2.6, respectively; all *p* < 0.001). Acquisition times increased from DWI(b1500) (107 ± 9 s) to DWI(b4500) (329 ± 26 s). Cohen κ for PI-RADS score agreement was good to moderate (DWI(b2500): 0.87 [confidence interval 0.81, 0.94]; DWI(b3500): 0.75 [0.65, 0.84]; DWI(4500): 0.61 [0.49, 0.72]).

**Conclusion:**

Acquired ultrahigh gradient DWI sequences with ultrahigh *b-*values in prostate MRI had worse image quality than standard *b-*values, while PI-RADS agreement between DWI(b1500) and DWI(b2500) was good. However, diagnostic estimates for clinically significant prostate carcinoma remained limited due to a small biopsy sample size (50/107 patients).

**Relevance statement:**

Ultrahigh *b-*value DWI showed no improved diagnostic performance in comparison to standard *b-*value DWI regarding the identification of potential prostate cancer. Ultrahigh *b-*value should not replace standard high *b-*values (1,500 s/mm²) for imaging workup of patients with suspicion for prostate cancer.

**Key Points:**

- *Acquired ultrahigh b-values (b2500–4500) using ultrahigh gradients of up to 140 T/m were utilized for prostate DWI*.
- *Both, overall image quality and diagnostic confidence decreased from good for DWI(b1500) to non-diagnostic for DWI(b4500)*.
- *PI-RADS agreement between DWI(b1500) and DWI(b2500) was good, while it was only moderate between DWI(b1500) and DWI(b4500)*.

**Graphical Abstract:**

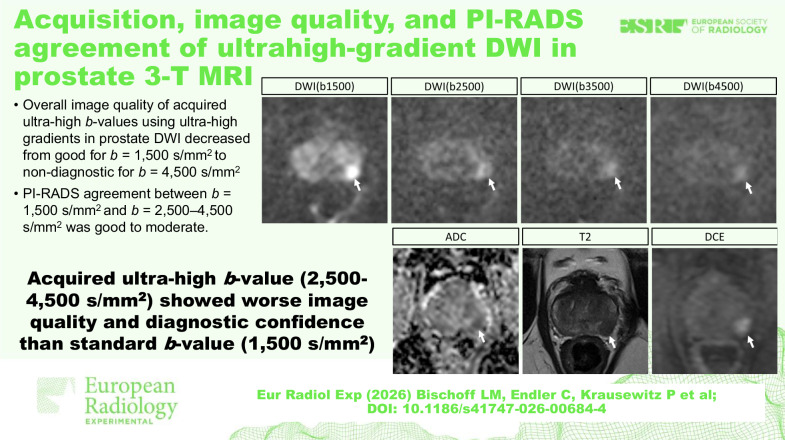

## Background

Diffusion-weighted imaging (DWI) sequences have an indispensable role in multiparametric magnetic resonance imaging (mpMRI) of the prostate due to their value for assessment of especially the peripheral zone [1, 2]. A high quality of these sequences is mandatory for lesion detection and subsequent successful MRI fusion biopsies to ensure targeted histologic evaluation of suspicious lesions [3, 4]. In the current Prostate Imaging Reporting and Data System (PI-RADS) recommendations, two distinct DWI sequences are recommended, including both a low *b-*value sequence (0‒100 s/mm^2^ and 800‒1,000 s/mm^2^) to calculate the apparent diffusion coefficient map and a high *b-*value sequence (> 1,400 s/mm^2^) to ensure better discrimination between benign and malignant lesions [1].

Different approaches to enhance these sequences have been developed in recent years, most notably by applying deep learning techniques for the removal of background noise [5, 6] or using advanced diffusion models [7]. However, while the former is increasingly used for various applications due to its convenient implementation on various MRI scanners [8, 9], the actual acquisition of ultrahigh *b-*values in clinical routine still remains challenging due to limited gradient strengths in a clinical setting, leading to long acquisition times and low signal-to-noise ratios.

To overcome this hardware-related limitation, studies have investigated the usability of computed high *b-*values. In general, DWI sequences with computed high *b-*values use extrapolations from lower *b-*values and thus maintain their main advantage of a higher signal-to-noise ratio. Acquired ultrahigh *b-*value sequences, however, are directly influenced by the molecular tissue structure and thus may be better at depicting real pathophysiological effects [10]. Thus, one can derive the hypothesis that acquired ultrahigh *b-*values could better discriminate between clinically significant and insignificant prostate carcinoma. However, Grant et al [11] could show equal lesion visibility in computed and acquired *b-*values up to 2,000 s/mm^2^, while another study could show higher lesion detectability at computed *b-*values of 2,000 and 3,000 s/mm^2^ [12].

Recent developments in gradient technology in the Human Connectome Project led to the implementation of ultrahigh potential gradient strengths (500 mT/m) and slew rates (600 T/m/s), and thus allowed acquisition of higher *b-*values due to a reduction of diffusion times [13, 14]. While these gradients have not explicitly been evaluated in prostate MRI, they enabled significantly better delineation of tissue microstructure in the human brain, for instance, through the measurement of the axon diameter [15, 16]. However, these specific MRI gradients were only used in a research setting in healthy volunteers. Recently, a new clinical 3-T MRI system with ultrahigh gradient strengths of up to 200 mT/m was approved to bridge the gap to applications in real patients.

Therefore, we utilized maximum available gradient strengths to acquire ultrahigh *b-*values of 1,500, 2,500, 3,500, and 4,500 s/mm^2^ and compared them with special regard to delineation of suspicious lesions and the apparent signal-to-noise (aSNR) and contrast-to-noise ratio (aCNR). A second objective was to compare the PI-RADS scores of these ultrahigh *b-*values compared to the reference standard of 1,500 s/mm².

## Materials and methods

### Study population

The institutional review committee of the University Hospital Bonn approved this prospective study, and the study was conducted according to the Declaration of Helsinki. Male participants with clinical suspicion of prostate cancer were included after written informed consent between November 2023 and February 2024. Inclusion criteria included either an elevated prostate-specific antigen of > 4 ng/mL, a suspicious digital rectal exam, and/or a suspicious transrectal US exam. Exclusion criteria included general MRI contraindications at 3 T (*e.g*., non-MRI compatible cardiac pacemaker), contraindications for administration of gadolinium-containing contrast media (*e.g*., prior severe allergic reaction), severe claustrophobia, and incomplete acquisition of MRI images.

### Image acquisition

MRI was performed using a 3-T MAGNETOM Cima.X scanner (Siemens Healthineers) and an 18-channel anterior phased array coil. The maximum employable gradient strength was 200 mT/m, the maximum slew rate 200 T/m/s. One mL hyoscine butylbromide (Butylscopolamin 20 mg/mL, Panpharma) was administered intravenously prior to the exam to reduce bowel peristalsis, 0.1 mmol/kg body weight of Gadoteric acid (Clariscan, GE Healthcare) for contrast-enhanced T1-weighted and dynamic contrast-enhanced sequences. The standard mpMRI protocol of the prostate, consisting of: multiplanar T2-weighted turbo spin-echo sequences; one low *b-*value DWI sequence (0/800 s/mm^2^) for calculation of the apparent diffusion coefficient map; one high *b-*value DWI sequence (1,500 s/mm^2^); one axial T1-weighted sequence pre and post contrast media administration; and one dynamic contrast-enhanced sequence (Time-resolved angiography With Stochastic Trajectories‒TWIST).

Additionally, DWI sequences with ultrahigh *b-*values of 2,500, 3,500, and 4,500 s/mm^2^ were acquired prior to contrast administration. All DWI sequences were acquired using echo-planar imaging with zonally-magnified oblique multislice acquisition‒ZOOM. For this sequence type, the maximum gradient strength was limited to 140 mT/m. Although the scanner has a gradient strength of up to 200 mT/m, these would further lower echo and repetition times and subsequently pose a risk for inadvertent cardiac stimulation. To prevent such events, the scanner software limits the maximum gradient strength in DWI ZOOM echo-planar imaging sequences by balancing minimum echo times and maximum gradient strength. Further increase of gradient strength (*i.e*., manually) is restricted by the software. A vendor-specific deep learning reconstruction algorithm for denoising and resolution upscaling (Deep Resolve Boost, Siemens Healthineers) was applied. Specific acquisition parameters of all sequences, including the actual employed gradient strengths, are listed in Table 1.

**Table 1:** Acquisition parameters

	DWI (b0/800)	DWI (b1500)	DWI (b2500)	DWI (b3500)	DWI (b4500)	T2w TSE	T2w TSE	T2w TSE	T1w TSE	T1w GRE DCE
Orientation	Axial	Axial	Axial	Axial	Axial	Axial	Sagittal	Coronal	Axial	3D
Acquisition Matrix	46 × 90	46 × 90	46 × 90	46 × 90	46 × 90	384 × 384	384 × 384	384 × 384	210 × 352	112 × 160
Field of view (mm^2^)	102 × 200	102 × 200	102 × 200	102 × 200	102 × 200	200 × 200	200 × 200	200 × 200	255 × 320	200 × 200
Spatial resolution (acquired) (mm)	2.2 × 2.2	2.2 × 2.2	2.2 × 2.2	2.2 × 2.2	2.2 × 2.2	0.5 × 0.5	0.5 × 0.5	0.5 × 0.5	1.2 × 0.9	1.8 × 1.3
Spatial resolution (reconstructed) (mm)	1.1 × 1.1	1.1 × 1.1	1.1 × 1.1	1.1 × 1.1	1.1 × 1.1	0.3 × 0.3	0.3 × 0.3	0.3 × 0.3	0.9 × 0.9	0.6 × 0.6
Slice thickness (mm)	3	3	3	3	3	3	3	3	2.5	3
Standard slice number (n)	26	26	26	26	26	26	24	25	88	24
Echo time (ms)	54	57	64	65	63	96	107	107	2.46	1.5
Repetition time (ms)	3,200	3,200	3,200	3,700	4,400	8,560	6,990	7,130	5.51	4.41
Flip angle (degree)	90	90	90	90	90	90	90	90	10	15
Averages	2 (b0), 8 (b800)	7	12	15	17	1	1	1	1	1
Diffusion gradients	4 directions (4-Scan Trace)	4 directions (4-Scan Trace)	4 directions (4-Scan Trace)	4 directions (4-Scan Trace)	4 directions (4-Scan Trace)	-	-	-	-	-
Gradient strength (mT/m)	98	111	116	129	140					
Bandwidth (Hz/pixel)	1,684	1,684	1,634	1,634	1,634	200	200	200	592	679
Acquisition time (s)	117	104	168	238	318	105	99	101	70	203
Temporal resolution (s)	-	-	-	-	-	-	-	-	-	4.22
Fat suppression	Fat-sat	Fat-sat	Fat-sat	Fat-sat	Fat-sat	-	-	-	Dixon	-
DL denoising	+	+	+	+	+	+	+	+	-	-
DL resolution upscaling	+	+	+	+	+	+	+	+	-	-

*DCE* Dynamic contrast enhanced, *DL* Deep learning, *DWI* Diffusion-weighted imaging, *GRE* Gradient echo, *T1w* T1-weighted, *T2w* T2-weighted, *TSE* Turbo spin-echo

### Qualitative image analysis

Two radiologists with 4 (L.M.B.) and 13 years (J.A.L.) of experience in prostate MRI rated all DWI sequences (b0/800, b1500, b2500, b3500, and b4500) on a Likert scale from 1 (non-diagnostic) to 5 (excellent) in the qualitative categories artifacts, image sharpness, lesion conspicuity, overall image quality, and diagnostic confidence.

Likert scores for artifacts were assigned as follows: 1, non-diagnostic due to substantial artifacts; 2, bad due to considerable artifacts; 3, moderate due to mild artifacts; 4, good due to minimal artifacts; 5, excellent due to no artifacts. Likert scores for image sharpness, lesion conspicuity, and overall image quality were assigned as follows: 1, severely blurred anatomical structures; 2, highly blurred anatomical structures; 3, fairly blurred anatomical structures; 4, minimal blurred anatomical structures; 5, absent blurring of anatomical structures. Likert scores for diagnostic confidence were assigned as follows: 1, no diagnostic confidence; 2, highly impaired diagnostic confidence; 3, limited diagnostic confidence; 4, satisfactory diagnostic confidence; 5, high diagnostic confidence. For the artifact ratings, all different types were considered, including movement artifacts, wrap-around artifacts, and susceptibility artifacts at rectal air-tissue interfaces. Readers were blinded to clinical data and the *b-*value of DWI sequences to prevent rater bias.

### Quantitative image analysis

Signal intensity (SI) was measured in the normal peripheral zone of the prostate and in the obturator internal muscle using a region of interest size of 15 and 30 mm², respectively. Additionally, in exams that were graded with at least a PI-RADS score of 3 during initial clinical reading, the SI of corresponding lesions was measured with a region of interest size of 15 mm². Subsequently, the apparent signal-to-noise ratio (aSNR) and the apparent contrast-to-noise ratio (aCNR) of all DWI sequences for both the normal peripheral zone and suspicious lesions were calculated as previously described [8]:

$${{{\rm{aSNR}}}}={{{{\rm{SI}}}}}_{{{{\rm{peripheral}}}}\; {{{\rm{zone}}}}}/{{{\rm{Standard\; deviation}}}}{{{{\rm{SI}}}}}_{{{{\rm{muscle}}}}}$$

$${{{\rm{aCNR}}}}=({{{{\rm{SI}}}}}_{{{{\rm{peripheral}}}}\; {{{\rm{zone}}}}}{{\mbox{-}}}{{{{\rm{SI}}}}}_{{{{\rm{muscle}}}}})/{{{\rm{Standard}}}}\; {{{\rm{deviation}}}}{{{{\rm{SI}}}}}_{{{{\rm{muscle}}}}}$$

It should be noted that these measurements only reflect apparent and not true signal-to-noise and contrast-to-noise ratios, as this would necessitate advanced analysis of raw data beyond the scope of this study [17].

### PI-RADS assessment

PI-RADS scores were assessed separately by three experienced radiologists with 4 (L.M.B.), 13 (J.A.L.), and 8 years (C.E.) of experience in prostate MRI according to ESUR/ESUI [18]. Readers were blinded to personal and clinical data, especially anamnestic information and values of the prostate-specific antigen. Each reader initially assigned a PI-RADS score to each participant, reading the full mpMRI protocol with inclusion of the DWI(b1500). Additionally, further PI-RADS readings were conducted using the ultrahigh *b-*value DWI(b2500), DWI(b3500), and DWI(4500) with a washout period of two months between each reading.

### Histopathological evaluation

Patients with PI-RADS 3–5 lesions underwent targeted MRI-fusion biopsy. Additionally, patients with PI-RADS 2 lesions that had a prostate-specific antigen density of > 0.15 ng/mL/cm³ underwent systematic MRI-fusion biopsy. Clinically significant prostate carcinoma was defined as an International Society of Uropathology (ISUP) score of ≥ 2. MRI to biopsy time intervals were noted. Biopsy results were stratified into three groups (no carcinoma, all carcinomas, carcinomas with an ISUP score of > 2) and correlated with PI-RADS ratings, where a PI-RADS score of ≤ 2 was considered as negative and a PI-RADS score of > 2 as positive.

### Statistical analysis

SPSS (Version 27, IBM Corp., Armonk, USA) and R (Version 4.5.2, R Foundation for Statistical Computing, Vienna, Austria) with RStudio (Version 2025.09.2) were used for statistical analysis. Due to the explorational nature of the study, the sample size was chosen to match previous studies investigating new techniques in prostate MRI [8, 10]. Continuous variables are shown as mean ± standard deviation, ordinal data as median and interquartile range (IQR), and dichotomous data as absolute percentages. A cumulative link mixed-effects model with the sequence and rater as fixed effects and the patients as random effects was used for testing the statistical significance of qualitative image parameters. Repeated measures ANOVA with *post hoc* Bonferroni test was used for testing the statistical significance of aSNR and aCNR. The *p*-values were adjusted with Bonferroni correction for multiple testing. PI-RADS agreement between DWI sequences was assessed by Cohen κ, while interreader reproducibility of PI-RADS scores was assessed by calculation of Fleiss κ. Similarly, interreader reproducibility of qualitative ratings was assessed using Cohen κ. Cohen and Fleiss κ were interpreted as follows: ≤ 0.5 = poor, 0.5–0.75 = moderate, 0.75–0.9 = good; > 0.9 = excellent. A *p*-value of < 0.05 was considered statistically significant.

## Results

### Clinical characteristics of participants

**Fig. 1:**
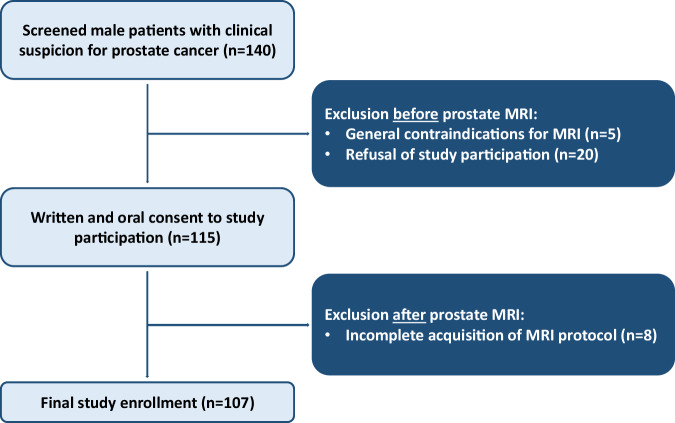
Flowchart of enrolled participants in the study

In total, 107 participants, aged 67 ± 8 years (mean ± standard deviation) were included after exclusion of 20 participants due to refusal of participation in the study, 5 due to general MRI contraindications, and 8 participants due to incomplete acquisition of DWI sequences (flow-chart in Fig. 1). The prostate specific antigen was elevated (> 4 ng/mL) in 90% (96/107) of participants, while 16% (17/107) had a suspicious digital rectal exam, and 9% (10/107) had a suspicious transrectal ultrasound. Of 107 participants, 26 (24%) had already undergone prostate biopsy prior to the MRI (Table 2). Following prostate MRI, 47% (50/107) underwent subsequent prostate biopsy, with confirmed prostate cancer in 58% (29/50). Median time between MRI and biopsy was 49 days (IQR: 37–66]. ISUP score ranged between 1 and 5 with a median score of 2 (IQR: 1–2] (Table S1).

**Table 2:** Clinical characteristics of enrolled participants

Variable	Value
No. of participants	107
Age (years)	67 ± 8
Prostate-specific antigen (ng/mL)	8.3 ± 6.3
Suspicious digital rectal exam	17 (16)
Suspicious transrectal ultrasound	10 (9)
Prior biopsy	26 (24)
PI-QUAL score	3 [IQR: 3–3]
PI-RADS score	
1	0 (0)
2	48 (45)
3	21 (20)
4	25 (23)
5	13 (12)

Continuous data is reported as mean ± standard deviation, dichotomous data as number of participants with percentages in parentheses

*IQR* Interquartile range, *PI-RADS* Prostate imaging reporting and data system

### Qualitative image analysis

Most artifacts were found in the DWI(b4500) (median score of both raters: 3 [IQR: 3–4]). The fewest artifacts were found in the DWI(b0/800) (median score of both raters: 4 [IQR: 4–4]; *p* < 0.001) and DWI(1500) (median score of both raters: 4 [IQR: 4–4]; *p* < 0.001).

Image sharpness was lowest in the DWI(b4500) (median score of both raters: 1 [IQR: 1–1]). The highest image sharpness was found in the DWI(b0/800) (median score rater 1: 4 [IQR: 4–4]; rater 2: 4 [IQR: 4–5]; *p* < 0.001).

The worst lesion conspicuity was found in the DWI(b4500) (median score of both raters: 2 [IQR: 1–2]). The highest lesion conspicuity was found in the DWI(b1500) (median score of both raters: 5 [IQR: 4–5]; *p* < 0.001).

The worst overall image quality was found in the DWI(b4500) (median score for rater 1: 1 [IQR: 1–2]; rater 2: 1 [IQR: 1–1]). The best overall image quality was found in the DWI(b0/800) (median score of both raters: 4 [IQR: 4–4]; *p* < 0.001) and DWI(b1500) (median score of both raters: 4 [IQR: 4–4]; *p* < 0.001). Of the three ultrahigh *b-*value DWI sequences (b2500, b3500, and b4500), the DWI(b2500) had the best overall image quality for both raters (median score of both raters: 3 [IQR: 2–3]).

The worst diagnostic confidence was found in the DWI(b4500) (median score of both raters: 1 [IQR: 1–2]). The best diagnostic confidence was found in the DWI(b1500) (median score for rater 1: 4.5 [IQR: 4–5]; rater 2: 5 [IQR: 4–5]; *p* < 0.001).

**Fig. 2:**
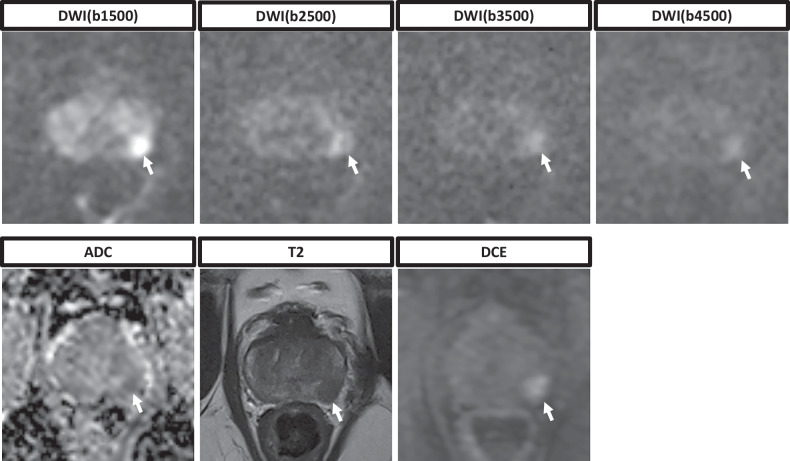
Representative images of a 61-year-old patient. The patient presented with an elevated prostate-specific antigen of 4.27 ng/mL. The whole multiparametric MRI protocol is shown, consisting of T2-weighted sequences, DWI sequences with calculation of the ADC map, and DCE sequences. The suspicious lesion in the posterolateral peripheral zone can be clearly delineated in the DWI(b1500), whereas both contrast and signal-to-noise ratio decrease in the DWI(b2500), DWI(b3500), and DWI(b4500). All readers gave a PI-RADS score of 4 for both readings that included either the DWI(b1500) or DWI(b2500). ADC, Apparent diffusion coefficient; DCE, Dynamic contrast enhanced; DWI, Diffusion-weighted imaging; MRI, Magnetic resonance imaging; PI-RADS, Prostate imaging reporting and data system

**Fig. 3:**
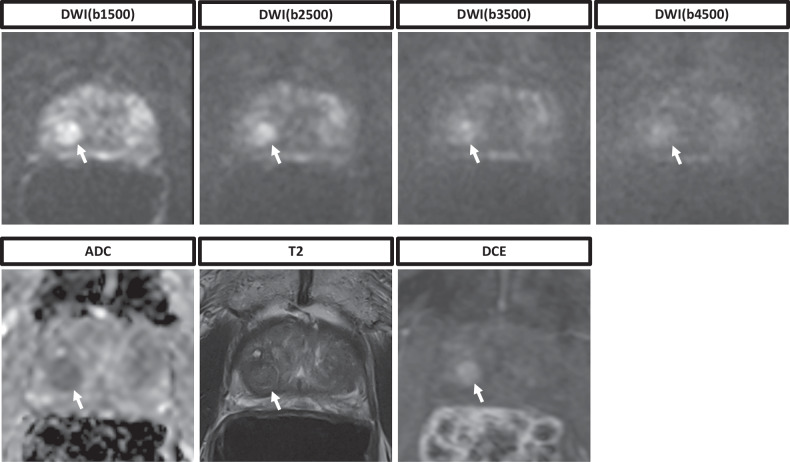
Representative images of a 79-year-old patient. The patient presented with a suspicious digital rectal exam while having a normal prostate-specific antigen of 2.5 ng/mL. The whole multiparametric MRI protocol is shown, consisting of T2-weighted sequences, DWI sequences with calculation of the ADC map, and DCE sequences. The heterogeneous lesion in the posterior transition zone shows a mostly encapsulated nodule that initially was rated by all readers with a PI-RADS score of 3 due to the additional diffusion restriction in both reading protocols that included either the DWI(b1500) or DWI(b2500). However, the diagnostic confidence declined with higher *b-*values. ADC, Apparent diffusion coefficient; DCE, Dynamic contrast enhanced; DWI, Diffusion-weighted imaging; MRI, Magnetic resonance imaging; PI-RADS, Prostate Imaging Reporting and Data System

**Fig. 4:**
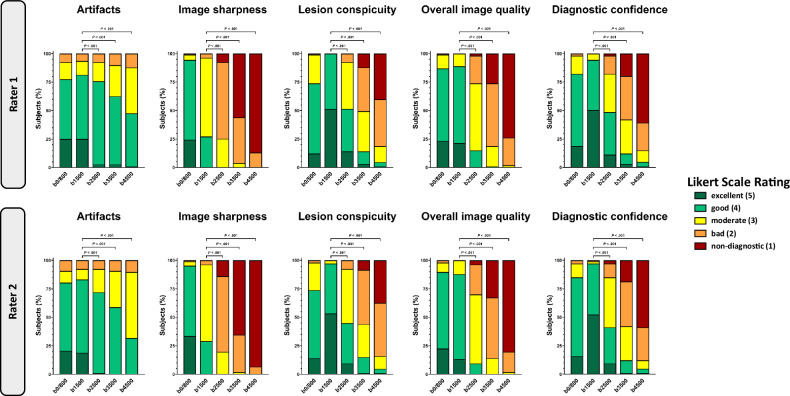
Qualitative image analysis. Stacked bar charts show Likert scale scores for the qualitative image analysis of DWI sequences stratified by *b-*value in the qualitative categories artifacts, image sharpness, lesion conspicuity, overall image quality, and diagnostic confidence. Overall, the high *b-*value DWI(b1500) was superior to the ultrahigh *b-*value DWI(b2500), DWI(b3500), and DWI(b4500) in each qualitative category (*p* < 0.001). A cumulative link mixed-effects model was used for the calculation of *p*-values. Only *p*-values between ultrahigh *b-*value sequences (DWI(b2500), DWI(b3500), DWI(b4500)) and the high *b-*value sequence DWI(b1500) are displayed. DWI, Diffusion-weighted imaging

Inter-reader reproducibility was good (Cohen κ, 0.87 [95% CI: 0.86, 0.88]). Representative images are shown in Figs. 2 and 3. For a full display of ratings, see Table 3 and Fig. 4.

**Table 3:** Qualitative analysis of DWI sequences

	Rater	DWI(b0/800)	DWI(b1500)	DWI(b2500)	DWI(b3500)	DWI(b4500)
Artifacts	1	4 [IQR: 4–4]^c,d,e^	4 [IQR: 4–4]^c,d,e^	4 [IQR: 4–4]^a,b,d,e^	4 [IQR: 3–4]^a,b,c,e^	3 [IQR: 3–4]^a,b,c,d^
	2	4 [IQR: 4–4]^c,d,e^	4 [IQR: 4–4]^c,d,e^	4 [IQR: 3–4]^a,b,d,e^	4 [IQR: 3–4]^a,b,c,e^	3 [IQR: 3–4]^a,b,c,d^
Image sharpness	1	4 [IQR: 4–4]^b,c,d,e^	3 [IQR: 3–4]^a,c,d,e^	2 [IQR: 2–2]^a,b,d,e^	1 [IQR: 1–2]^a,b,c,e^	1 [IQR: 1–1]^a,b,c,d^
	2	4 [IQR: 4–5]^b,c,d,e^	3 [IQR: 3–4]^a,c,d,e^	2 [IQR: 2–2]^a,b,d,e^	1 [IQR: 1–2]^a,b,c,e^	1 [IQR: 1–1]^a,b,c,d^
Lesion conspicuity	1	4 [IQR: 3–4]^b,c,d,e^	5 [IQR: 4–5]^a,c,d,e^	4 [IQR: 3–4]^a,b,d,e^	2 [IQR: 2–3]^a,b,c,e^	2 [IQR: 1–2]^a,b,c,d^
	2	4 [IQR: 3–4]^b,c,d,e^	5 [IQR: 4–5]^a,c,d,e^	3 [IQR: 3–4]^a,b,d,e^	2 [IQR: 2–3]^a,b,c,e^	2 [IQR: 1–2]^a,b,c,d^
Overall image quality	1	4 [IQR: 4–4]^c,d,e^	4 [IQR: 4–4]^c,d,e^	3 [IQR: 2–3]^a,b,d,e^	2 [IQR: 1–2]^a,b,c,e^	1 [IQR: 1–2]^a,b,c,d^
	2	4 [IQR: 4–4]^c,d,e^	4 [IQR: 4–4]^c,d,e^	3 [IQR: 2–3]^a,b,d,e^	2 [IQR: 1–2]^a,b,c,e^	1 [IQR: 1–1]^a,b,c,d^
Diagnostic confidence	1	4 [IQR: 4–4]^b,c,d,e^	4,5 [IQR: 4–5]^a,c,d,e^	3 [IQR: 3–4]^a,b,d,e^	2 [IQR: 2–3]^a,b,c,e^	1 [IQR: 1–2]^a,b,c,d^
	2	4 [IQR: 4–4]^b,c,d,e^	5 [IQR: 4–5]^a,c,d,e^	3 [IQR: 3–4]^a,b,d,e^	2 [IQR: 2–3]^a,b,c,e^	1 [IQR: 1–2]^a,b,c,d^

*DWI* Diffusion-weighted imaging

^a^ *Post hoc* Bonferroni test *p* < 0.050 *versus* DWI(b0/800)

^b^ *Post hoc* Bonferroni test *p* < 0.050 *versus* DWI(b1500)

^c^ *Post hoc* Bonferroni test *p* < 0.050 *versus* DWI(b2500)

^d^ *Post hoc* Bonferroni test *p* < 0.050 *versus* DWI(b3500)

^e^ *Post hoc* Bonferroni test *p* < 0.050 *versus* DWI(b4500)

### Quantitative image analysis

**Fig. 5:**
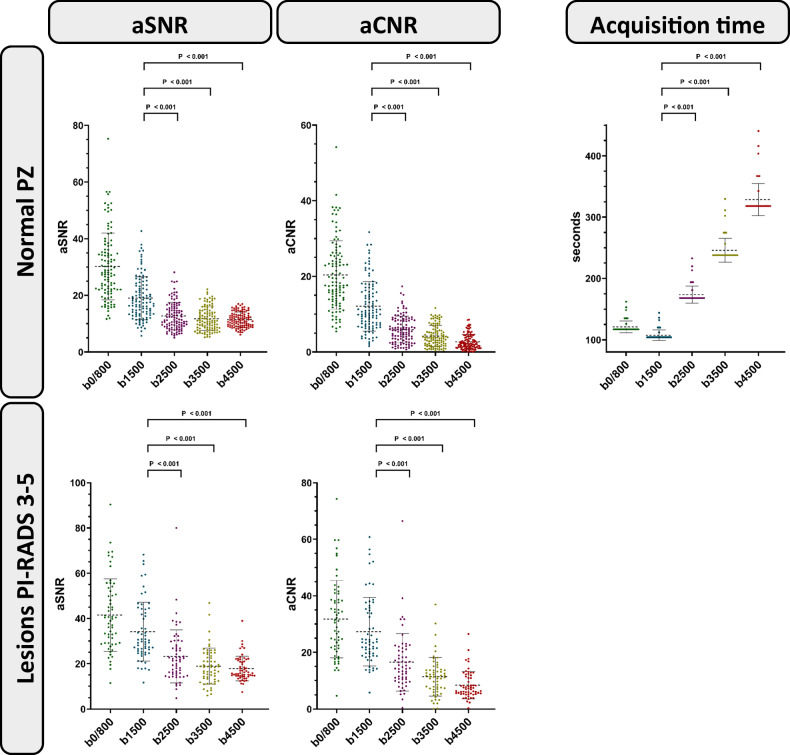
Quantitative image analysis. Dot plots show quantitative analysis of DWI sequences stratified by *b-*value in the categories aSNR and aCNR for both the normal PZ and suspicious lesions, and acquisition duration. Both mean aSNR and aCNR decrease continuously from the low *b-*value DWI to the ultrahigh *b-*value DWI. While the DWI(b1500) had the lowest acquisition time with 107 ± 9 s, the DWI(b4500) had the highest acquisition time with 328 ± 26 s. Horizontal dotted lines represent the mean. Repeated measures analysis of variance with the *post hoc* Bonferroni test was used for the calculation of *p*-values. *p*-values between ultrahigh *b-*value sequences (DWI(b2500), DWI(b3500), DWI(b4500)) and the high *b-*value sequence DWI(b1500) are displayed. aCNR, Apparent contrast-to-noise ratio; aSNR, Apparent signal-to-noise ratio; DWI, Diffusion-weighted imaging; PI-RADS, Prostate Imaging Reporting and Data System; PZ Peripheral zone

For the normal peripheral zone, the aSNR declined from DWI(b0/800) to DWI(b4500) (30.2 ± 11.8 *versus* 11.4 ± 2.6; *p* < 0.001). Similarly, the aCNR declined from DWI(b0/800) to DWI(b4500) (20.4 ± 9.1 *versus* 2.7 ± 2.0; *p* < 0.001). For suspicious lesions in general, the aSNR declined from DWI(b0/800) to DWI(b4500) (41.5 ± 16.0 *versus* 17.8 ± 5.5; *p* < 0.001). Similarly, the aCNR declined from DWI(b0/800) to DWI(b4500) (31.8 ± 13.7 *versus* 8.4 ± 4.8; *p* < 0.001). For suspicious lesions in the peripheral zone, the aSNR declined from DWI(b0/800) to DWI(b4500) (41.7 ± 16.8 *versus* 17.7 ± 5.6; *p* < 0.001). Similarly, the aCNR declined from DWI(b0/800) to DWI(b4500) (32.0 ± 14.6 *versus* 8.2 ± 4.9; *p* < 0.001). For suspicious lesions in the transition zone, the aSNR declined from DWI(b0/800) to DWI(b4500) (42.3 ± 12.7 *versus* 17.8 ± 4.4; *p* < 0.001). Similarly, the aCNR declined from DWI(b0/800) to DWI(b4500) (32.0 ± 14.6 *versus* 8.2 ± 4.9; *p* < 0.001). The mean acquisition times of the DWI(b0/800), DWI(b1500), DWI(b2500), DWI(b3500) and DWI(b4500) were 121 ± 10 s, 107 ± 9 s, 174 ± 14 s, 246 ± 20 s and 329 ± 26 s, respectively (Fig. 5).

### Comparison of PI-RADS scores

Of all ultrahigh *b-*value DWI sequences, the DWI(b2500) had the best agreement with the standard DWI(b1500), which was good for all raters (Cohen κ reader 1, 0.87 [95% CI: 0.81, 0.94]; reader 2, 0.88 [95% CI: 0.81, 0.95]; reader 3, 0.89 [95% CI: 0.82, 0.96]) with similar values after stratification for lesions in the peripheral and transition zone (Table 4). For readers 1, 2, and 3, PI-RADS scores for the DWI(b2500) were lower than for the DWI(b1500) in 14% (15/107), 10% (11/107) and 9% (10/107) of participants. In contrast, they were higher in 0% (0/107), 2% (2/107) and 1% (1/107) of participants, respectively (Table S2). Consequently, readers 1, 2, and 3 changed a PI-RADS score of ≥ 3 in the DWI(b1500) to a PI-RADS score of 2 in the DWI(b2500) in 7% (8/107), 6% (6/107) and 8% (9/107) of participants, respectively. In these, a subsequent biopsy was performed in 63% (5/8), 50% (3/6) and 67% (6/9), and prostate cancer was confirmed in 40% (2/5), 100% (3/3) and 100% (3/3), respectively.

**Table 4:** PI-RADS score agreement between DWI(b1500) and the ultrahigh *b-*value sequences DWI(b2500), DWI(b3500) and DWI(4500)

	Overall	Peripheral zone	Transition zone
DWI(b2500)			
Reader 1	0.87 (0.81, 0.94)	0.89 (0.83, 0.96)	0.83 (0.70, 0.97)
Reader 2	0.88 (0.81, 0.95)	0.89 (0.82, 0.96)	0.89 (0.79, 1.0)
Reader 3	0.89 (0.82, 0.96)	0.88 (0.81, 0.96)	0.95 (0.87, 1.0)
DWI(b3500)			
Reader 1	0.75 (0.65, 0.84)	0.76 (0.66, 0.85)	0.83 (0.70, 0.97)
Reader 2	0.77 (0.67, 0.86)	0.79 (0.69, 0.88)	0.83 (0.70, 0.97)
Reader 3	0.76 (0.67, 0.85)	0.76 (0.67, 0.86)	0.86 (0.74, 0.99)
DWI(b4500)			
Reader 1	0.61 (0.49, 0.72)	0.62 (0.50, 0.74)	0.77 (0.61, 0.93)
Reader 2	0.65 (0.53, 0.76)	0.67 (0.55, 0.79)	0.79 (0.63, 0.95)
Reader 3	0.62 (0.51, 0.74)	0.63 (0.51, 0.75)	0.80 (0.65, 0.95)

Data is reported as Cohen κ with 95% confidence intervals in parentheses

*DWI* Diffusion-weighted imaging, *PI-RADS* Prostate imaging reporting and data system

Of 29 participants with subsequent histological confirmation of prostate cancer, readers 1, 2, and 3 assigned a PI-RADS score of ≥ 3 in DWI(b1500) in 90% (26/29), 90% (26/29), and 93% (27/29), respectively. Opposed to this, readers 1, 2, and 3 assigned a PI-RADS score of ≥ 3 in DWI(b4500) in 62% (18/29), 66% (19/29), and 69% (20/29) of participants, respectively.

Similarly, of 18 participants with subsequent histological confirmation of clinically significant prostate cancer, readers 1, 2, and 3 assigned a PI-RADS score of ≥ 3 in DWI(b1500) in 94% (17/18), 94% (17/18), and 94% (17/18), respectively. Opposed to this, readers 1, 2, and 3 assigned a PI-RADS score of ≥ 3 in DWI(b4500) in 72% (13/18), 78% (14/18), and 78% (14/18) of participants, respectively (Table 5).

**Table 5:** Correlation of histopathological results with PI-RADS scores dependent on *b-*values of DWI sequences

	No carcinoma and PI-RADS ≤ 2	All carcinomas and PI-RADS > 2	Carcinoma ISUP score ≥ 2 and PI-RADS > 2
Reader 1			
DWI(b1500)	33% (7/21)	90% (26/29)	94% (17/18)
DWI(b2500)	48% (10/21)	83% (24/29)	94% (17/18)
DWI(b3500)	48% (10/21)	72% (21/29)	83% (15/18)
DWI(b4500)	62% (13/21)	62% (18/29)	72% (13/18)
Reader 2			
DWI(b1500)	33% (7/21)	90% (26/29)	94% (17/18)
DWI(b2500)	33% (7/21)	79% (23/29)	94% (17/18)
DWI(b3500)	52% (11/21)	69% (20/29)	83% (15/18)
DWI(b4500)	62% (13/21)	66% (19/29)	78% (14/18)
Reader 3			
DWI(b1500)	33% (7/21)	93% (27/29)	94% (17/18)
DWI(b2500)	43% (9/21)	83% (24/29)	89% (16/18)
DWI(b3500)	48% (10/21)	76% (22/29)	78% (14/18)
DWI(b4500)	62% (13/21)	69% (20/29)	78% (14/18)

Percentages of patients with specified histopathological results and PI-RADS scores for the acquired DWI values are shown

*DWI* Diffusion-weighted imaging, *ISUP* International Society of Pathology, *PI-RADS* Prostate imaging reporting and data system

Overall inter-reader agreement was good (Fleiss κ, 0.87 [95% CI: 0.84, 0.91]).

## Discussion

In this study, we aimed to use a novel clinical MRI scanner with a maximum gradient strength of 200 mT/m to acquire DWI sequences with ultrahigh *b-*values of up to 4,500 s/mm² and compare them to low (0/800 s/mm²) and high (1,500 s/mm²) *b-*value DWI sequences with focus on image quality and impact on PI-RADS scores. Compared to the standard DWI(b1500), all ultrahigh *b-*value DWI sequences had decreased overall image quality, while the acquisition times of DWI(b2500), DWI(b3500), and DWI(b4500) were increased by 63%, 130%, and 207%, respectively. Additionally, the prediction accuracy for histologically confirmed prostate cancer declined with higher *b-*values. Thus, ultrahigh *b-*values were not able to replace standard *b-*values in terms of image quality, cancer detection, and acquisition efficiency.

The acquisition of DWI sequences with high *b-*values > 1,000 s/mm² has already been implemented in the current PI-RADS 2.1 recommendations due to their high diagnostic performance compared to lower *b-*values [1]. However, additional studies investigating ultrahigh *b-*values of 2,000 s/mm² showed mixed results. While most studies indicated superior or equal image quality and diagnostic performance of ultrahigh *b-*values of 2,000 s/mm² compared to high *b-*values of 1,000 s/mm² [19–21], others showed lower sensitivity and specificity [22]. Our results confirm those findings, demonstrating an overall good PI-RADS agreement between DWI(b1500) and DWI(b2500). However, PI-RADS agreement of DWI(b1500) with higher *b-*values (DWI(b3500) and DWI(b4500)) was significantly worse. Additionally, the number of PI-RADS 3‒5 scores for lesions that subsequently were histologically confirmed as prostate cancer decreased from 90–93% in the DWI(b1500) to 62–69% in the DWI(b4500), indicating a lower diagnostic performance of measured ultrahigh *b-*values of ≥ 2500 s/mm². Furthermore, we found DWI sequences with ultrahigh *b-*values of ≥ 2500 s/mm² to have less diagnostic confidence, which was mostly related to the decreased signal.

While the actual acquisition of *b-*values > 2500 s/mm² has until recently been limited due to gradient strength limitations, their synthetic calculation has already been investigated. However, in contrast to extrapolating low *b-*values for the synthesis of high *b-*values, the actual acquisition of higher *b-*values is influenced by physical effects such as intracellular diffusion. Most studies found the optimal height of both synthetic and acquired *b-*values range to lay from 1,500 to 2,500 s/mm² [20, 23–25]; only a single study suggested an ultrahigh *b-*value of 3,200 s/mm^2^ [26]. Similarly, we observed results in line with the previously mentioned publications regarding the acquisition of ultrahigh *b-*values, which led to a significant decrease in contrast, and ultimately to a lower conspicuity of lesions due to frequent insufficient differentiation from background noise. Further development of other diffusion models like VERDICT or IVIM may be better capable of detecting suspicious prostatic lesions than acquired ultrahigh *b-*value acquisitions [7, 27].

Another critical point to emphasize is the high increase in acquisition time, ranging from 1 min and 57 ± 9 s for DWI(b1500) to 5 min and 28 ± 26 s for DWI(b4500). While this not only led to an increase in movement artifacts, it also counteracts the current trend towards faster acquisition protocols [28–30].

Our study has limitations. First, not all participants underwent subsequent biopsy due to the high amount of PI-RADS 2 lesions. According to the PI-RADS recommendations and recent study results, biopsy is not recommended for these lesions [1, 31]. As a consequence, histopathological verification was not available for all reported lesions, and the overall calculation of sensitivity and specificity is missing. This might have promoted a verification bias in our study results. Future studies with more detailed histopathological validation may clarify these details. Second, the detection accuracy for the presence of clinically significant prostate cancer would be highly interesting. While this study already assessed some of these patients, their number was too low to make reliable conclusions. However, prior studies could not show advantages of computed high *b-*value DWI sequences, and the impact of actual acquired DWI sequences on the detection of clinically significant prostate cancer is still insufficiently studied [20, 32]. Third, although we applied several different variations of parameters, especially adjustments of repetition and echo time, the maximal gradient strength in this body application was limited by the software of the clinical scanner to 140 mT/m to avoid adverse cardiac stimulation. Thus, it remains unclear, how actual gradient strengths of 200 mT/m may influence the image quality of ultrahigh *b-*value DWI sequences and the assessment of PI-RADS scores. Fourth, qualitative and quantitative evaluations may have been influenced by the use of deep learning reconstructions. However, these reconstructions were applied in all DWI acquisitions, the effect on the final reconstructions is likely considered to be similar. The same applies to stronger technical equipment like body coils with more channels that would likely improve the signal-to-noise ratio of all acquired sequences.

In conclusion, the use of a novel 3-T MRI scanner with ultrahigh gradient DWI and *b-*values of up to 4,500 s/mm² led to a significant increase in acquisition times, a decrease in lesion conspicuity, and a substantial decrease in diagnostic confidence under our acquisition conditions. Although this technique has proven to be beneficial in different imaging fields such as brain imaging, the naturally higher gradient limitations render it unviable for this body application. Overall, *b-*values around 1,500 s/mm² for DWI sequences still provide the best diagnostic value in terms of image quality and acquisition time.

## Supplementary information

**Additional file 1:** **Table S1** Biopsy results. **Table S2** Contingency tables of PI-RADS ratings for DWI(b1500) and DWI(b2500) for all readers.

## Data Availability

The datasets used and/or analyzed during the current study are available from the corresponding author on reasonable request.

## Abbreviations

aCNR: Apparent contrast-to-noise ratio
aSNR: Apparent signal-to-noise ratio
DWI: Diffusion-weighted imaging
IQR: Interquartile range
ISUP: International Society of Uropathology
mpMRI: Multiparametric MRI
PI-RADS: Prostate imaging reporting and data system
SI: Signal intensity
