# Supplementary material for: Acquisition, image quality, and PI-RADS agreement of ultrahigh-gradient DWI in prostate 3-T MRI
Source: Eur Radiol Exp. 2026 Feb 23;10:17. doi: 10.1186/s41747-026-00684-4 (PMC12929734; doi:10.1186/s41747-026-00684-4)
Supplement: Supplementary file 1 — Additional file 1: Table S1 Biopsy results. Table S2 Contingency tables of PI-RADS ratings for DWI(b1500) and DWI(b2500) for all readers. [file 41747_2026_684_MOESM1_ESM.pdf]

Acquisition, image quality, and PI-RADS agreement of ultrahigh-gradient DWI in prostate 3-T MRI

ELECTRONIC SUPPLEMENTARY MATERIAL

Table S1 Biopsy results

PI-RADS score	Performed fusion biopsy (*n* =50)	Confirmed malignancy (*n* = 29)	ISUP grade
1 (*n* = 0)	-	-	-
2 (*n* = 48)	8 (17)	3 (38)	Grade 1: 2 (67)<br>Grade 5: 1 (33
3 (*n* = 21)	11 (52)	3 (27)	Grade 1: 1 (33)<br>Grade 2: 2 (67)
4 (*n* = 25)	21 (84)	14 (67)	Grade 1: 5 (36)<br>Grade 2: 7 (50)<br>Grade 4: 1 (7)<br>Grade 5: 1 (7)
5 (*n* = 13)	10 (77)	9 (90)	Grade 1: 3 (33)<br>Grade 2: 5 (56)<br>Grade 5: 1 (11)

Dichotomous data is reported as number of participants with percentages in parentheses. *ISUP* International Society of Pathology, *PI-RADS* Prostate Imaging Reporting and Data System.

Table S2 Contingency tables of PI-RADS ratings for DWI(b1500) and DWI(b2500) for all readers

**Reader 1**			**b1500**				
			***n* = 0**	***n* = 51**	***n* = 15**	***n* = 29**	***n* = 12**
**PI-RADS scores *(n* =**			**1**	**2**	**3**	**4**	**5**
	***n* = 0**	**1**	0	0	0	0	0
	***n* = 59**	**2**	0	51	7	1	0
**b2500**	***n* = 15**	**3**	0	0	8	7	0
	***n* = 21**	**4**	0	0	0	21	0
	***n* = 12**	**5**	0	0	0	0	12

**Reader 2**			**b1500**				
			***n* = 0**	***n* = 52**	***n* = 18**	***n* = 25**	***n* = 12**
**PI-RADS scores *(n* = *107)***			**1**	**2**	**3**	**4**	**5**
	***n* = 0**	**1**	0	0	0	0	0
	***n* = 58**	**2**	0	52	4	2	0
**b2500**	***n* = 17**	**3**	0	0	12	5	0
	***n* = 20**	**4**	0	0	2	18	0
	***n* = 12**	**5**	0	0	0	0	12

**Reader 3**			**b1500**				
			***n* = 0**	***n* = 50**	***n* = 20**	***n* = 25**	***n* = 12**
**PI-RADS scores *(n* = *107)***			**1**	**2**	**3**	**4**	**5**
	***n* = 0**	**1**	0	0	0	0	0
	***n* = 58**	**2**	0	49	6	3	0
b2500	***n* = 16**	**3**	0	1	14	1	0
	***n* = 21**	**4**	0	0	0	21	0
	***n* = 12**	**5**	0	0	0	0	12

Data are absolute numbers. *bpMRI* Biparametric MRI, *mpMRI* Multiparametric MRI, *PI-RADS* Prostate Imaging Reporting and Data System.
